# Association Between Nighttime Discharge from the Intensive Care Unit and Hospital Mortality: A Multi-Center Retrospective Cohort Study

**DOI:** 10.1186/s12913-015-1044-4

**Published:** 2015-09-14

**Authors:** Luciano CP Azevedo, Ivens A. de Souza, David A. Zygun, Henry T. Stelfox, Sean M. Bagshaw

**Affiliations:** Division of Critical Care Medicine, Faculty of Medicine and Dentistry, University of Alberta, 2-124E Clinical Sciences Building, 8440-122 Street, Edmonton, AB T6G 2B7 Canada; Department of Critical Care Medicine, Alberta Health Services, Edmonton Zone, 2-124E Clinical Sciences Building, 8440-122 Street, Edmonton, AB T6G 2B7 Canada; Research and Education Institute, Hospital Sírio-Libanês, São Paulo, Brazil; Emergency Medicine Department ICU, University of São Paulo, São Paulo, Brazil; Departments of Critical Care Medicine, Medicine and Community Health Sciences, Institute for Public Health, University of Calgary, Calgary, Canada

## Abstract

**Background:**

We aimed to determine the impact of nighttime discharge from the intensive care unit (ICU) to the ward on hospital mortality and readmission rates in consecutive critically ill patients admitted to five Canadian ICUs. We hypothesized that hospital mortality and readmission rates would be higher for patients discharged after hours compared with discharge during the day.

**Methods:**

A multi-center retrospective cohort study was carried out at five hospitals in Edmonton, Canada, between July 2002 and December 2009. Nighttime discharge was defined as discharge from the ICU occurring between 07:00 pm and 07:59 am. Logistic regression analysis was used to explore the associations between nighttime discharge and outcomes.

**Results:**

Of 19,622 patients discharged alive from the ICU, 3,505 (17.9 %) discharges occurred during nighttime. Nighttime discharge occurred more commonly among medical than surgical patients (19.9 % vs. 13.8 %, *P* < 0.001) and among those with more comorbid conditions, compared with daytime discharged patients. Crude hospital mortality (11.8 % versus 8.8 %, *P* < 0.001) was greater for nighttime discharged as compared to daytime discharged patients. In a multivariable analysis, after adjustment for comorbidities, diagnosis and source of admission, nighttime discharge remains associated with higher mortality (odds ratio [OR] 1.29; 95 % CI, 1.14 to 1.46, *P* < 0.001). This finding was robust in two sensitivity analyses examining discharges occurring between 00:00 am and 04:59 am (OR 1.28; 1.12–1.47; *P* < 0.001) and for those who died within 48 h of ICU discharge without readmission (OR 1.24; 1.07–1.42, *P* = 0.002). There was no difference in ICU readmission for nighttime compared with daytime discharges (7.4 % vs. 6.9 %, *p* = 0.26). However, rates were higher for nighttime discharges in community compared with tertiary hospitals (7.7 % vs. 5.7 %, *P* = 0.023).

**Conclusions:**

In a large integrated health region, 1 in 5 ICU patients are discharged at nighttime, a factor with increasing occurrence during our study and shown to be independently associated with higher hospital mortality.

**Electronic supplementary material:**

The online version of this article (doi:10.1186/s12913-015-1044-4) contains supplementary material, which is available to authorized users.

## Background

Intensive care unit (ICU) beds are a valuable resource for management of patients with life-threatening critical illness. In recent years, even though the number of ICU beds has increased, the demand for these beds has also increased in a proportion often exceeding supply [1]. As such, ICUs often operate at high occupancy rates, contributing to refused or delayed admissions and cancellations of elective surgeries due to bed unavailability [2]. Increased pressure for ICU beds also contributes to premature patient discharge and suboptimal transition of care [3]. These patients are at increased risk for preventable adverse events, such as death or unplanned ICU readmission [4]. Selected studies have suggested that ICU discharge on weekends and at nighttime are associated with increased risk for less favorable outcomes including mortality [3, 5–7], but findings have been inconsistent [1, 8]. Reasons for these discrepant findings possibly relate to different health systems’ organizations with diverse services delivery and staffing patterns, variation in case-mix and variable definitions of nighttime discharge.

We hypothesized that hospital mortality and unplanned ICU readmission rates would be higher for critically ill patients discharged at nighttime from the ICU compared with those discharged during the day. Accordingly, we performed a retrospective study to describe nighttime discharge from ICU to the ward and hospital mortality and ICU readmissions in an integrated Canadian health region.

## Methods

The study was approved by the regional Health Research Ethics Board at the University of Alberta, Edmonton, Canada prior to commencement. The Clinical Department of Critical Care Medicine for Alberta Health Services, Edmonton Zone (inclusive of each ICU contributing to this study) provided authorization for access to administrative data for this study. The need for written informed consent was waived. The reporting of this study follows the STROBE guideline (available at: http://www.strobe-statement.org/).

### Study design and setting

This was a multi-center retrospective observational cohort study performed at all adult hospitals in Edmonton, Canada between July 1, 2002 and December 31, 2009. In 2009, Edmonton had a population of 782,000 and a metropolitan population of 1,110,000 [9]. The three community hospitals included the Grey Nuns Community Hospital (GNH) (8 beds – 1 daytime intensivist, 1 nighttime intensivist), Misericordia Community Hospital (MIS) (6 ICU plus 4 high dependency beds – 1 daytime intensivist, 1 nighttime intensivist), and Sturgeon General Hospital (SGH) (5 beds – 1 daytime intensivist, 1 nighttime intensivist). These three hospitals all have mixed medical/surgical ICUs. The GNH is the regional referral center for all major vascular surgery. The two tertiary hospitals included the Royal Alexandria Hospital (RAH) (22 beds – 2 daytime intensivist teams, 1 nighttime intensivist), and University of Alberta Hospital (UAH) (30 beds – 3 daytime intensivist teams and an intensivist-led rapid response team, 1 nighttime intensivist). Both of these tertiary hospitals have mixed medical/surgical/trauma ICUs. The RAH is a level II trauma center. The UAH is a level I trauma center and the regional referral for all non-cardiac solid-organ transplantation. All study ICUs operate by a closed care model. All study ICUs had in-house coverage by clinical associates or resident trainees, and the nighttime intensivist was available but not necessarily in-house.

### Study population

All consecutive adult (age ≥ 18 years) patients admitted to the five hospitals’ participating ICUs who were discharged alive to the ward from the ICU were eligible. If a patient had multiple ICU admissions during a single hospitalization, only the first admission was included for primary analysis. Patients with missing data on vital status were excluded.

### Study definitions

*Nighttime discharge* was defined as discharge from ICU occurring between 07:00 pm and 07:59 am. *Daytime discharge* was defined as a discharge from ICU occurring between 08:00 am and 06:59 pm. *Weekend discharge* was defined as discharge from ICU occurring between 07:00 pm on Friday and 07:59 am on Monday. *ICU readmission* was defined as return to ICU within the index hospitalization. We further evaluated ICU readmission by whether it occurred within 72 h of the index ICU discharge based on the rationale that this event may be more related to residual critical illness rather than another discrete event. *ICU admission source* was classified as emergency department (ED), operating room/emergency post-operative status, operating room/elective post-operative status, transfer from other institutions, or in-hospital ward transfer. Severity of illness was defined according to the Acute Physiology and Chronic Health Evaluation (APACHE) II score [10]. Chronic organ dysfunction was defined according to the definitions used to calculate the chronic health component of the APACHE II score [10]. *Hepatic failure* was defined as having documented cirrhosis by histology or elevated bilirubin and INR attributed to liver disease prior to the index hospitalization associated with ICU admission. *Immunosuppression* was defined as having received cytotoxic medication and/or steroids within the 7 days preceding ICU admission. *Chronic respiratory disease* was defined as documented need for home oxygen therapy and/or severe exercise restriction prior to the index hospitalization associated with ICU admission. *Chronic kidney disease* was defined as chronic dialysis therapy prior to the index hospitalization associated with ICU admission. *Hematologic cancer* was defined as having pathologically confirmed lymphoma, leukemia or multiple myeloma. *Congestive heart failure* was defined as having symptoms at minimal exertion prior to the index hospitalization associated with ICU admission. *Surgical status* was defined as having had an operative procedure within 7 days of ICU admission.

### Data sources

We utilized an ICU-specific clinical and administrative database maintained by the regional Division of Critical Care Medicine, termed the Minimal Data Set (MDS) database. Trained data coordinators abstracted demographic, diagnostic, clinical, physiologic and outcome data for each discrete patient admission to ICU to the five participating hospitals in Edmonton [9]. We extracted data on patient demographics, ICU admission source, ICU discharge time, post-operative status, co-morbid conditions, primary ICU admission diagnoses, necessity for mechanical ventilation, APACHE II score, hospital length of stay and in-hospital mortality.

### Statistical analysis

The primary exposure of interest was time of ICU discharge (nighttime vs. daytime). The primary outcome measure was in-hospital mortality. The secondary outcome measures were hospital length of stay and readmissions to the ICU. Two additional a priori planned sensitivity analysis were performed. The first omitted patients discharged alive from ICU who died within 48 h. The second restricted the analysis to nighttime ICU discharge occurring between 00:00 am and 04:59 am [3]. Descriptive normally or near normally distributed data are reported as means with standard deviations (SD) and compared by Student’s t-test. Non-normally distributed continuous data are reported as medians with inter-quartile ranges (IQR) and were compared by Mann Whitney U test. Categorical variables were compared using the Chi-squared test. We evaluated for trends in nighttime ICU discharge and readmission rate by using straight-line regression of the natural logarithm of the discharge rate, with calendar year as the independent variable. Estimated annual percentage change was equal to [100 × (exp(b)-1] where b represents the slope of the regression. If the estimate annual percentage change is statistically greater than zero, then the incidence rate has an increased trend over the study period [11]. Customized multi-variable logistic regression models with in-hospital mortality and ICU readmission as dependent variables and nighttime discharge as an independent variable were created, that adjusted for demographics, co-morbidity, APACHE II score; use of mechanical ventilation; ICU length-of-stay, surgical status; admission source; and primary diagnostic category (i.e., cardiovascular, gastrointestinal, neurologic and metabolic), study year and type of hospital. This analysis was replicated using a mixed-methods approach with a random effect for hospital. Data are reported as odds ratios (OR) with 95 % confidence intervals (CI). Data were evaluated for multi-collinearity. Model discrimination was assessed by the area under the receiver operating characteristic curve (AUC) and fit by the Pearson goodness-of-fit (GoF) test, respectively. All statistical analyses were two-sided and *p* < 0.05 was considered significant. Statistical analyses were conducted using Intercooled Stata Release 10 (Stata Corp, College Station, TX).

## Results

There were 24,829 ICU admissions to the five ICUs during the study period. After excluding 1,642 due to repeat admissions and 399 admissions due to missing data on vital status, the study cohort comprised of 22,788 unique ICU admissions (93.3 %). The 2 tertiary hospitals contributed the majority of discharges (*n* = 13,924; 71.0 %) during the study period, with fewer occurring in the 3 community hospitals (*n* = 5,698; 29 %). Nighttime discharges were more common in these hospitals when compared with the community hospitals (18.9 % vs. 15.3 %; *P* < 0.001).

**Fig. 1:**
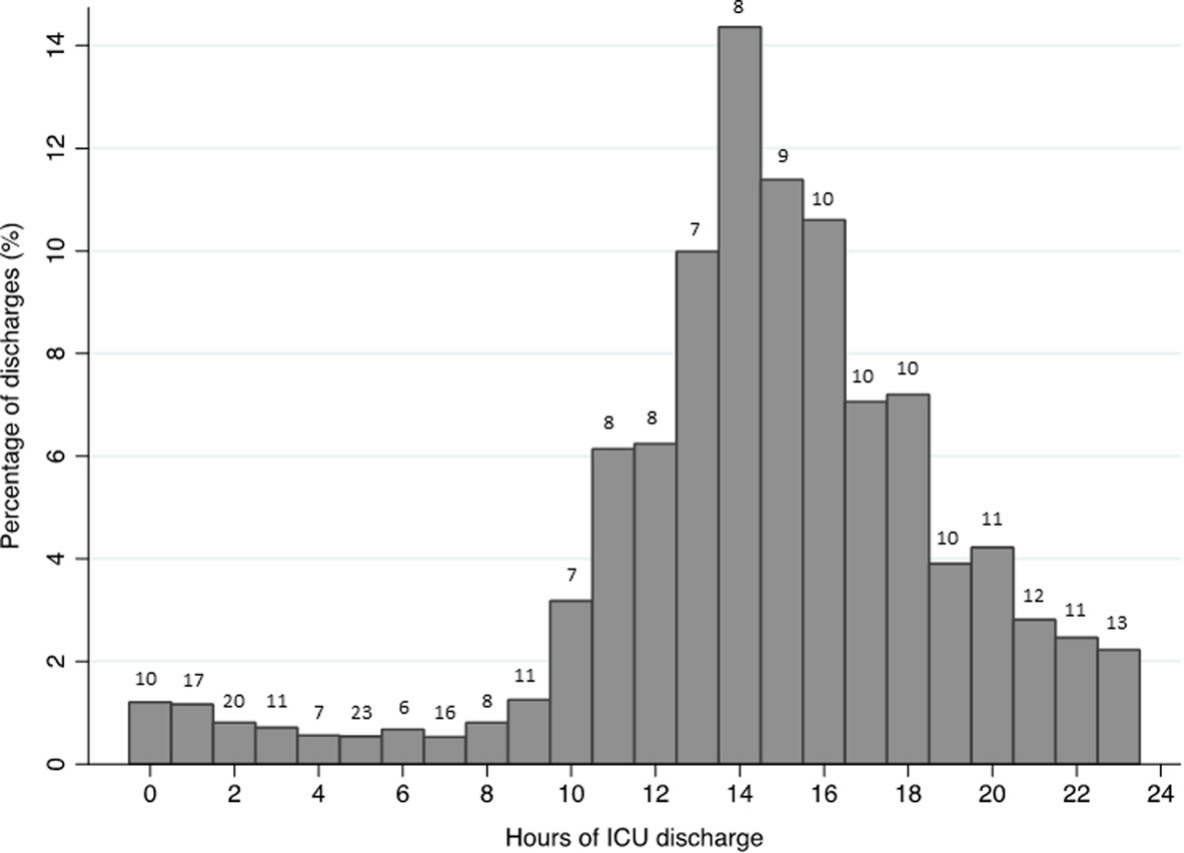
Histogram showing the distribution of hours of ICU discharge for the study population as well as the unadjusted mortality rates distributed according to hour of discharge

Survival to ICU discharge and ward transfer was 86.1 % (*n* = 19,622). Of these, 3,505 (17.9 %) discharges occurred during nighttime, from 07:00 pm to 07:59 am, and 16,117 (82.1 %) during daytime, from 08:00 am to 06:59 pm (Fig. 1).

### Factors associated with nighttime discharge

Patients discharged at nighttime were more commonly admitted from the ward or ED compared to daytime discharged patients, who were more commonly admitted from the operating room (Table 1). Nighttime discharges occurred more commonly among medical compared with surgical patients (19.9 % vs. 13.8 %; *P* < 0.001). Patients discharged at nighttime had more comorbid illness, higher admission APACHE II scores and were more likely to have received mechanical ventilation. Nighttime discharged patients were less likely to have gastrointestinal and cardiovascular diagnoses and more likely to have neurologic diagnoses when compared to daytime discharges, respectively.

**Table 1:** Baseline characteristics of the study cohort stratified by time of ICU discharge

Characteristics	Total	Nighttime discharges	Daytime discharges	*P* value
	n: 19,622	*n* = 3,505 (17.9 %)	*n* = 16,117 (82.1 %)	
Male sex, n (%)	11,357 (57.9)	1,986 (56.7)	9,371 (58.1)	0.11
Age (years) (mean [SD])	57.5 (18.0)	57.2 (17.9)	57.5 (18.0)	0.30
Apache II score (mean [SD])	19.4 (7.5)	20.1 (7.6)	19.3 (7.4)	<0.001
Medical admission, n (%)	13,018 (66.3)	2,596 (74.1)	10,422 (64.7)	<0.001
Post-operative, n (%)	6,604 (33.7)	909 (25.9)	5,695 (35.3)	<0.001
Comorbid illness, n (%)				
None	15,180 (77.4)	2,662 (76.0)	12,518 (77.7)	0.027
One	3,927 (20.0)	730 (20.8)	3,197 (19.8)	0.19
Two or more	515 (2.6)	113 (3.2)	402 (2.5)	0.014
Comorbid condition, n (%)				
Hepatic failure	873 (4.5)	150 (4.3)	723 (4.5)	0.59
Immunosuppression	981 (5.0)	207 (5.9)	774 (4.8)	0.007
Chronic lung disease	1,355 (6.9)	228 (6.5)	1,127 (7.0)	0.30
Chronic renal failure	613 (3.1)	159 (4.5)	454 (2.8)	<0.001
Hematologic cancer	314 (1.6)	63 (1.8)	251 (1.6)	0.31
Metastatic cancer	465 (2.4)	83 (2.4)	382 (2.4)	0.99
Congestive heart failure	293 (1.5)	53 (1.5)	240 (1.5)	0.92
AIDS	82 (0.4)	16 (0.5)	66 (0.4)	0.69
Primary diagnosis, n (%)				
Respiratory	5,940 (30.3)	1,099 (31.4)	4,841 (30.0)	0.12
Gastrointestinal	3,342 (17.0)	520 (14.8)	2,822 (17.5)	<0.001
Cardiovascular	2,822 (14.4)	448 (12.8)	2,374 (14.7)	0.003
Sepsis	1,548 (7.9)	295 (8.4)	1,253 (7.8)	0.20
Trauma	1,699 (8.7)	312 (8.9)	1,387 (8.6)	0.57
Metabolic	1,640 (8.4)	315 (9.0)	1,325 (8.2)	0.14
Neurologic	1,385 (7.1)	293 (8.4)	1,092 (6.8)	0.001
Renal	626 (3.2)	121 (3.5)	505 (3.1)	0.33
Other	620 (3.2)	102 (2.9)	518 (3.2)	0.35
Mechanical ventilation, n (%)	14,349 (73.1)	2,641 (75.3)	11,708 (72.6)	0.001
Source of admission, n (%)				
Emergency department	6,370 (32.5)	1,275 (36.4)	5,095 (31.6)	<0.001
Operating room - Elective	3,165 (16.1)	401 (11.4)	2,764 (17.1)	<0.001
Operating room – Emergency	3,438 (17.5)	508 (14.5)	2,930 (18.2)	<0.001
Other hospital	3,140 (16.0)	603 (17.2)	2,537 (15.7)	0.032
Ward	3,509 (17.9)	718 (20.5)	2,791 (17.3)	<0.001
Year of admission, n (%)				
2002/2003	3852 (19.6)	577 (14.9)	3275 (75.1)	<0.01*
2004/2005	4929 (25.1)	801 (16.2)	4128 (83.8)	
2006/2007	5400 (27.5)	1007 (18.6)	4393 (81.4)	
2008/2009	5441 (27.7)	1120 (20.6)	4321 (79.4)	
ICU LOS, (d) (med [IQR])	3 (1–7)	3 (1–7)	3 (2–7)	0.09
Source of discharge, n (%)				
Ward	17,935 (91.4)	3,199 (91.3)	14,736 (91.4)	0.75
Acute care	979 (5.0)	260 (7.4)	719 (4.5)	<0.001
Home	585 (3.0)	31 (0.9)	554 (3.4)	<0.001
Left without authorization	75 (0.4)	11 (0.3)	64 (0.4)	0.46
Chronic care	48 (0.2)	4 (0.1)	44 (0.3)	0.08
Weekend discharges	4,676 (23.8)	779 (16.7)	3,897 (83.3)	0.014
Weekday discharges	14,946 (76.2)	2,726 (18.2)	12,220 (81.8)	0.014

Legend: AIDS: Acquired Immune Deficiency Syndrome; ICU: Intensive Care Unit; LOS: Length of stay

*Linear regression

Nighttime discharges were more common during weekdays compared to weekend days (18.2 % vs. 16.7 %; *P* = 0.014). There was an increasing trend for nighttime discharges during the study period of 10.9 % per period (95 % CI, 7.8–14.0 %; *P* < 0.01) (Table 1). This represented a relative increase of 27.2 % over the entire study period. Further analysis of these nighttime discharge trends by hospital type showed that the increasing trend was driven by nighttime discharges in tertiary hospitals and not community hospitals (Additional file 1: Table e-1).

### Association of nighttime discharge and mortality

Crude hospital mortality (11.8 % vs. 8.8 %, *P* < 0.001) was greater for ICU patients discharged at nighttime compared with daytime (Table 2). After adjustment, nighttime discharge remained associated with higher odds of hospital death (aOR 1.29; 95 % CI, 1.14 to 1.46, *P* < 0.001) (Table 3). Additional independent risks for hospital mortality included higher admission APACHE II score, older age, presence of comorbid conditions, receipt of mechanical ventilation, being admitted from the ED or ward, being admitted to the ICU from 2006 onwards, longer ICU length of stay, and having gastrointestinal, cardiovascular and neurologic diagnoses (Table 3). In a further mixed-methods analysis, accounting for the random effect of each hospital site, the adjusted OR was similar (aOR 1.30; 95 % CI, 1.15–1.48, *P* < 0.001). There were no significant differences in hospital lengths of stay between nighttime and daytime discharges, even when discriminated by survival status (Table 2).

**Table 2:** Hospital mortality, length of stay and readmission rate stratified by ICU discharge time

Outcomes	Total	Nighttime discharges	Daytime discharges	*P* value
	n: 19,622	*n* = 3,505 (17.9 %)	*n* = 16,117 (82.1 %)	
Hospital mortality, n (%)				
All	1,837 (9.4)	412 (11.8)	1,425 (8.8)	<0.001
Post-operative admission	429 (6.5)	80 (8.8)	349 (6.1)	0.002
Emergency department admission	603 (9.5)	143 (11.2)	460 (9.0)	0.017
Other hospital admission	195 (6.2)	47 (7.8)	148 (5.8)	0.07
Ward admission	610 (17.4)	142 (19.8)	468 (16.8)	0.06
Community hospitals admissions	492 (8.6)	95 (10.9)	397 (8.2)	0.009
Tertiary hospitals admissions	1,345 (9.7)	317 (12.0)	1,028 (9.1)	<0.001
Hospital LOS survivors, (d)	15 (8–31)	15 (8–30)	14 (7–29)	0.12
Hospital LOS non-survivors, (d)	23 (11–45)	20 (11–43)	23 (12–45)	0.11
Post ICU Hospital LOS, (d)	9 (3–23)	10 (4–23)	10 (4–22)	0.06
Readmissions to the ICU, n (%)	1369 (7.0)	260 (7.4)	1,109 (6.9)	0.26
Within 72 hs after discharge	538 (2.7)	107 (3.0)	431 (2.7)	0.21
More than 72 h after discharge	831 (4.2)	153 (4.4)	678 (4.2)	0.67
Community hospitals admissions	343 (6.0)	67 (7.7)	276 (5.7)	0.023
Tertiary hospitals admissions	1,026 (7.4)	193 (7.3)	833 (7.4)	0.92

Legend: ICU: Intensive Care Unit; LOS: Length of stay (median [IQR])

**Table 3:** Summary of multivariable adjusted logistic regression analysis for hospital death among critically ill patients after ICU discharge

Predictor variables	Odds ratio (OR)	95 % CI	*P*-value
Discharge time			
Daytime	1.0	(reference)	
Nighttime	1.29	1.14–1.46	<0.001
Discharge day			
Weekday	1.0	(reference)	
Weekend	0.95	0.84–1.07	0.39
APACHE II score	1.06	1.05–1.07	<0.001
Age (per year)	1.04	1.04–1.04	<0.001
Burden of comorbidities			
None	1.0	(reference)	
Just one	1.40	1.24–1.58	<0.001
Two or more	1.47	1.13–1.91	0.004
Mechanical ventilation	1.19	1.03–1.37	0.018
Admission source			
Operating room	1.0	(reference)	
ED	1.77	1.50–2.08	<0.001
Other hospital	1.02	0.83–1.26	0.82
Ward	2.58	2.18–3.04	<0.001
Study year			
2002/2003	1.0	(reference)	
2004/2005	1.09	0.93–1.28	0.28
2006/2007	1.27	1.08–1.48	0.003
2008/2009	1.17	1.00–1.37	0.048
Hospital type			
Community	1.0	(reference)	
Tertiary	1.07	0.95–1.20	0.28
ICU stay (log)	1.28	1.20–1.37	<0.001
Admission diagnosis			
Respiratory	1.0	(reference)	
Gastrointestinal	1.88	1.59–2.21	<0.001
Cardiovascular	1.32	1.11–1.57	0.002
Sepsis	1.18	0.98–1.42	0.08
Trauma	0.85	0.63–1.15	0.29
Endo/metabolic	0.54	0.39–0.76	<0.001
Neurologic	2.20	1.80–2.68	<0.001
Renal	1.11	0.83–1.48	049
Other	1.77	1.27–2.47	0.001

Abbreviations: *OR* odds ratio, *ED* emergency department, *LOS*, length of stay

AuROC: 0.787 (0.777–0.797)

GoF test: 0.9904

### Association of nighttime discharge and ICU readmission

Overall, there was no significant difference in ICU readmission rate for patients discharged at nighttime compared with daytime (7.4 % vs. 6.9 %, *p* = 0.26) (Table 2). However, in community hospitals, readmission rate was higher for nighttime compared with daytime discharges (7.7 % versus 5.7 %, *P* = 0.023) (Table 2). Overall, there was no significant trend for increasing readmission rate during the study period (10.9 % per period; 95 % CI, -8.4 to 30.2 %) (Table 3). In multivariable analysis, independent risks for ICU readmission included higher illness severity, older age, comorbidity, admission after the year 2004, length of ICU stay, and having primary gastrointestinal/hepatic failure and sepsis diagnoses.

### Sensitivity analysis

In a sensitivity analysis, we omitted patients that died within 48 h after discharge from the ICU and were not readmitted, as this indirectly implied death was expected and they were discharged after having a change in their goals of care to palliation. This represented 391 patients (21 % of deaths). After exclusion of these patients, in multivariable analysis (Additional file 2: Table e-2), nighttime discharge remained significantly associated with hospital mortality (OR 1.24; 95 % CI, 1.07–1.42, *P* = 0.002). In a further sensitivity analysis, we examined the association of late night discharge between 00:00 a.m. and 04:59 a.m., based on the rationale that there is little justification for discharge at this time and that these discharges may be more likely to occur due to strain on ICU bed availability. In total, 551 patients (15.7 % of nighttime discharges; 2.8 % of total discharges) were discharged during this period. Late night discharge remained similarly associated with an increased risk of hospital mortality (OR 1.28; 95 % CI, 1.12–1.47, *p* < 0.001) (Additional file 3: Table e-3).

## Discussion

We performed a multi-center, retrospective cohort study to describe the association between nighttime discharge from ICU to the ward and hospital mortality and risk of ICU readmission.

### Summary of major findings

We found that approximately one in five patients are discharged from the ICU at nighttime. Patients discharged at night were more likely medical, had more comorbid disease, and were more severely ill at the time of ICU admission as compared to those discharged during the daytime hours. The number of nighttime discharges increased significantly in the two tertiary hospitals during the study period, indirectly suggesting that these ICUs have increasingly been confronted by strain on ICU capacity. Importantly, we showed that nighttime discharge was independently associated with a higher risk for hospital mortality, even when we excluded patients that died early after ICU discharge. Although nighttime discharge was not independently associated with ICU readmission, our data showed readmission rates were higher in community hospitals during the study period. This may represent another proxy measure of strain on ICU capacity. Patients who were older, burdened by greater comorbid disease, had higher admission illness severity, had primary hepatic or septic diagnoses and longer ICU stay were more likely to be readmitted.

### Comparison with previous studies

Prior investigations have evaluated the association between time of ICU discharge and outcomes [8, 12–15]. While most studies demonstrate that nighttime discharge is associated with unfavorable outcomes [7, 12–14], others have failed to confirm these findings [1, 8, 16]. In a landmark study from the ICNARC database in the United Kingdom, Goldfrad and Rowan showed that nighttime discharge from ICU increased risk for hospital mortality [3]. However, after adjustment for “premature discharge” in their analysis, the independent effect of nighttime discharge was lost [3], suggesting that the attributable risk for mortality was more related to the untimely discharge rather than the specific time of day. In a subsequent study of Finnish ICU patients, Uusaro et al failed to show “out-of-office” hour discharges (defined as those occurring from 1600 h to 0800 h) were associated with post-ICU mortality [8]. However, their definition for out-of-office hours was more liberal than ours and those of other studies. Hanane et al, in an analysis of three ICUs in a single hospital, also failed to show an association between nighttime discharge and mortality; however, nighttime discharge was associated with higher rates of ICU readmission and longer hospital lengths of stay [1]. Interestingly, these authors adjusted for patients’ preferences for “do-not-resuscitate” and site-specific characteristics such as the availability of ICU beds, which could have represented important confounders for the association of nighttime discharge and mortality [1]. Notably, nighttime discharged patients in this study had more comorbidities and higher disease severity [1], findings that were reproduced in our study and others [2, 3, 12, 17]. In general; however, the majority of studies that have examined the consequences of nighttime discharge have shown this to be an independent risk for mortality or unplanned readmission [1, 6, 7, 14]. Of note, the magnitude of this risk of death in our study (OR 1.29; 95 % CI, 1.14–1.46; Table 3) aligns with prior data [2, 3, 5, 17–19], and adds further confidence to the generalizability of this association.

Arguably, from the perspective of the patient, there is no logical reason to transition a critically ill patient from the ICU to the ward at nighttime. There are plausible explanations for why such nighttime discharges carry a higher risk of adverse outcomes for patients when compared to planned discharges during daytime hours. Nighttime discharges are often provoked by reduced ICU bed availability and the need to accommodate the next more severely ill patient [12]. These discharges may be unplanned, chaotic, less well coordinated and may negatively impact patient safety by predisposing to adverse events and medical errors, regardless of protocolized hand-over procedures and standardized transfer orders. Furthermore, these nighttime discharges are more likely to be premature, where patients are stepped down to a setting of lower intensity monitoring at a time of day when less resources are available [3]. Prior studies provide support for these hypotheses. Premature ICU discharge has been shown to be an independent risk for mortality [20]. Interestingly, a triage model to predict premature ICU discharge for “at-risk” patients was found to predict a 39 % reduction in mortality if these patients remained in the ICU for further 48 h [21].

High occupancy and reduced ICU bed availability at the time of discharge portends a higher risk for death or ICU readmission [4]. Moreover, the risk of early ICU readmission has been shown several-fold higher during periods of increased demand characterized by a high number of patient ICU admissions [22]. Increased risk of ICU readmission [23] and death [24] associated with admission to busy ICUs has similarly been confirmed. Although we did not have data on corresponding occupancy rates and measures of patient flow in our ICUs, based on prior data [3], we speculate that a significant proportion of nighttime discharges occur in response to increased demand for ICU beds. We believe this may be indirectly supported by the increasing trend for nighttime discharges, occurring primarily in tertiary hospitals (Table 3; Additional file 1: Table e-1); the trends for greater adjusted odds for mortality for nighttime discharges in the later years of the study, notably from 2006 onwards, and our sensitivity analyses. This trend for increasing nighttime discharges has similarly been described in other studies [5, 7], reinforcing the theory that a major driver for nighttime discharges is limited ICU bed availability. However, this finding is not universal. Recently, the Australian and New Zealand Intensive Care Society (ANZICS) reported no increase nighttime discharges over an 8-year period [19]. Whether this is explained by changes to policy, operations or expanded ICU capacity is uncertain. In another prospective multicenter study, patient-level factors at the time of ICU admission and discharge, including illness severity, need for complex therapy and the presence of orders for limitations in medical therapy, were integrated into an analysis of the association between nighttime discharge and hospital mortality. Both nighttime discharge rate (16.4 %) and hospital mortality (5.2 %) were lower than observed in our study. After adjusting for these factors, in particular limitations to medical therapy, nighttime discharge was no longer associated with hospital mortality (OR 1.16; 95 % CI, 0.89–1.53) [16]. While this study is clearly an important contribution by exposing the potential confounding influence of limits to medical therapy provided, it did not specifically adjudicate whether hospital deaths were “expected” in those with care limitations and, similar to our study, operations data on occupancy and patient flow were not available, which may impact its external validity.

### Study limitations

Our study has notable limitations. First, although our study involved 5 discrete hospital ICUs in a large integrated health region and utilized high-quality prospectively collected data, our findings may have limited generalizability, and due to its observational design, are potentially predisposed to bias and residual confounding. Specifically, our study does not enable a detailed evaluation of true demand on ICU resources, strain on capacity and operations that may influence the flow of patients and decisions to discharge at night. In addition, we recognize that the majority of ICU admissions were directed to the two larger tertiary institutions, where over time, nighttime discharges increased in frequency. On the other hand, our findings are importantly coherent with similar observational studies performed in varied health jurisdictions. Second, our findings may not be applicable to other health systems where ICU capacity issues are not as prevalent [25]. Third, we did not have data on what proportion of nighttime discharged patients were designated palliative and not for ICU readmission, which may have unduly influenced our results. However, we found our effect estimate for mortality robust in sensitivity analysis after excluding those patients that died within 48 h of ICU discharge and not readmitted, which may represent a surrogate for a change in goals of care. Furthermore, ICU readmission as a quality and performance measure and outcome may be limited due to lack of standardization in criteria, inability to adjudicate whether readmission was avoidable and whether readmission was attributable to residual issues related to critical illness, ward care, or other unmeasured factors (i.e., provider decision-making). Fourth, in our multi-variable analysis, we utilized the admission APACHE II score for adjustment which may not correlate with the clinical status at the time of ICU discharge. Finally, we did not have detailed information about ICU staffing, temporary bed closures, and nuanced hospital-wide operational or bed management characteristics across the included hospitals (other than knowing all ICUs have closed intensivist models) to integrate into our modeling.

### Areas of future research

Our findings, in the context of prior studies, suggest some patient-specific factors (i.e., case-mix, illness severity) may influence nighttime discharge and risk of adverse outcome; however, health system factors (i.e., ICU demand; ICU bed availability, hospital bed management priorities) likely exert a greater influence on the probability of nighttime discharge and risk of adverse outcome. The impact of differing adaptive models to ICU or ward care, quality of transition (i.e., hand off), and ICU outreach on reducing premature or unnecessary nighttime discharges or avoidable adverse events remains to be explored. Further high-quality and high-fidelity evaluative studies are needed to better examine the patient-specific and health services factors (i.e., operational models of patient flow) associated with nighttime discharges and risk of adverse events.

## Conclusions

In a large integrated Canadian health region, 1 in 5 critically ill patients are discharged from the ICU at nighttime. This phenomenon showed an increasing trend during the study in tertiary hospitals. Nighttime discharge was independently associated with increased in-hospital mortality, but not increased length of stay or ICU readmission across the entire sample. Increased likelihood of ICU readmission was observed in the subgroup of nighttime discharges occurring in community hospitals. Critically ill patients with greater burdens of comorbid illness and higher illness severity at ICU admission were at increased risk for death or ICU readmission following nighttime discharge. These data would suggest that nighttime discharge should be avoided whenever possible and that further evaluation incorporating patient, ICU and hospital level factors and operational management processes should be undertaken.

## Additional files

Additional file 1:
**Discharges and period of admission.** Percentage of discharges according to period of admission and type of hospital. (DOCX 14 kb) Additional file 2:
**Sensitivity analysis.** Multivariable logistic regression analysis showing the association of hospital death with nighttime/daytime discharges, weekend/weekday discharges, APACHE II score, age, burden of comorbidities, mechanical ventilation at admission, source of admission, study year, study site, log-transformation of ICU length of stay and admission diagnosis, after excluding patients (*n* = 391) with early (≤48 h) death after ICU discharge. (DOCX 16 kb) Additional file 3:
**Sensitivity analysis.** Multivariable logistic regression analysis showing the association of hospital death with late night/daytime and early night discharges, weekend/weekday discharges, APACHE II score, age, burden of comorbidities, mechanical ventilation at admission, source of admission, study year, study site, log-transformation of ICU length of stay and admission diagnosis. (DOCX 16 kb)

## Abbreviations

APACHE II: Acute Physiology and Chronic Health Evaluation II
AUR ROC: Area under the receiver operating characteristic curve
ANZICS: Australian and New Zealand Intensive Care Society
CI: Confidence interval
ED: Emergency Department
GNH: Grey Nuns Community Hospital
GoF: Pearson goodness-of-fit
ICU: Intensive Care Unit
IQR: Interquartile range
MIS: Misericordia Community Hospital
OR: Odds ratio
RAH: Royal Alexandria Hospital
SGH: Sturgeon General Hospital
UAH: University of Alberta Hospital
