# Supplementary material for: Association Between Nighttime Discharge from the Intensive Care Unit and Hospital Mortality: A Multi-Center Retrospective Cohort Study
Source: BMC Health Serv Res. 2015 Sep 14;15:378. doi: 10.1186/s12913-015-1044-4 (PMC4570509; doi:10.1186/s12913-015-1044-4)
Supplement: Additional file 1: — Discharges and period of admission. Percentage of discharges according to period of admission and type of hospital. (DOCX 14 kb) [file 12913_2015_1044_MOESM1_ESM.docx]

**Additional file 1**

***NIGHTTIME DISCHARGE FROM THE INTENSIVE CARE UNIT IS ASSOCIATED WITH INCREASED IN-HOSPITAL MORTALITY: A MULTI-CENTER RETROSPECTIVE COHORT STUDY***

Luciano C.P. Azevedo ^1,2,3^, Ivens A. de Souza ^1,2^, David A. Zygun^1^, Henry T. Stelfox^4^, Sean M. Bagshaw^1^

^1^Division of Critical Care Medicine, Faculty of Medicine and Dentistry, University of Alberta, 2-124E Clinical Sciences Building, 8440-122 Street, Edmonton, AB, T6G 2B7, Canada

^2^Research and Education Institute, Hospital Sírio-Libanês, São Paulo, Brazil

^3^Emergency Medicine Department ICU, University of São Paulo, Brazil.

^4^Department of Critical Care Medicine, Community Health Sciences, Faculty of Medicine, University of Calgary

**Table e-1.** Percentage of discharges according to period of admission and type of hospital.

|  | **Community**  **Hospitals** | | | **Tertiary Hospitals** | | |
| --- | --- | --- | --- | --- | --- | --- |
| **Year of admission, n (%)** | **Total Discharges**  **(n=5698)** | **Daytime discharges**  **(n=4828)** | **Nighttime discharges**  **(n=870)** | **Total Discharges**  **(n=13924)** | **Daytime discharges**  **(n=11289)** | **Nighttime discharges**  **(n=2635)** |
| 2002/2003 | 897(16) | 800 (89) | 97 (11) | 2955 (21) | 2475 (84) | 480 (16) |
| 2004/2005 | 1338 (23) | 1124 (84) | 214 (16) | 3591 (26) | 3004 (84) | 587 (16) |
| 2006/2007 | 1644 (29) | 1382 (84) | 262 (16) | 3756 (27) | 3011 (80) | 745 (20) |
| 2008/2009 | 1819 (32) | 1522 (84) | 297 (16) | 3622 (26) | 2799 (77) | 823 (23) |
| **P value** | --- | P=02 | | ----- | P<0.01 | |
