# Supplementary material for: Association Between Nighttime Discharge from the Intensive Care Unit and Hospital Mortality: A Multi-Center Retrospective Cohort Study
Source: BMC Health Serv Res. 2015 Sep 14;15:378. doi: 10.1186/s12913-015-1044-4 (PMC4570509; doi:10.1186/s12913-015-1044-4)
Supplement: Additional file 3: — Sensitivity analysis. Multivariable logistic regression analysis showing the association of hospital death with late night/daytime and early night discharges, weekend/weekday discharges, APACHE II score, age, burden of comorbidities, mechanical ventilation at admission, source of admission, study year, study site, log-transformation of ICU length of stay and admission diagnosis. (DOCX 16 kb) [file 12913_2015_1044_MOESM3_ESM.docx]

**Additional file 3**

***NIGHTTIME DISCHARGE FROM THE INTENSIVE CARE UNIT IS ASSOCIATED WITH INCREASED IN-HOSPITAL MORTALITY: A MULTI-CENTER RETROSPECTIVE COHORT STUDY***

Luciano C.P. Azevedo ^1,2,3^, Ivens A. de Souza ^1,2^, David A. Zygun^1^, Henry T. Stelfox^4^, Sean M. Bagshaw^1^

^1^Division of Critical Care Medicine, Faculty of Medicine and Dentistry, University of Alberta, 2-124E Clinical Sciences Building, 8440-122 Street, Edmonton, AB, T6G 2B7, Canada

^2^Research and Education Institute, Hospital Sírio-Libanês, São Paulo, Brazil

^3^Emergency Medicine Department ICU, University of São Paulo, Brazil.

^4^Department of Critical Care Medicine, Community Health Sciences, Faculty of Medicine, University of Calgary

**Table e-3 -** Multivariable logistic regression analysis showing the association of hospital death with late night (00:00 am – 04:00 am)/daytime and early night discharges, weekend/weekday discharges, APACHE II score, age, burden of comorbidities, mechanical ventilation at admission, source of admission, study year, study site, log-transformation of ICU length of stay and admission diagnosis.

| **Predictor Variables** | **OR** | **95% CI** | **p-value** |
| --- | --- | --- | --- |
| **Discharge Time** |  |  |  |
| Daytime/Early night | 1.0 |  |  |
| Late night | 1.28 | 1.12 - 1.47 | <0.001 |
| **Discharge Day** |  |  |  |
| Weekday | 1.0 |  |  |
| Weekend | 0.94 | 0.84 - 1.07 | 0.368 |
| **APACHE II score** | 1.06 | 1.05 – 1.07 | <0.001 |
| **Age (per year)** | 1.04 | 1.03 – 1.04 | <0.001 |
| **Burden of Comorbidities** |  |  |  |
| No comorbidity | 1.0 |  |  |
| Just one comorbidity | 1.40 | 1.24 – 1.58 | <0.001 |
| Two or more comorbidities | 1.48 | 1.13 – 1.91 | 0.004 |
| **Mechanical Ventilation** |  |  |  |
| No | 1.0 |  |  |
| Yes | 1.18 | 1.03 – 1.37 | 0.016 |
| **Source of admission** |  |  |  |
| Postoperative | 1.0 |  |  |
| ED | 1.79 | 1.52 – 2.11 | <0.001 |
| Other Hospital | 1.03 | 0.84– 1.27 | 0.722 |
| Ward | 2.60 | 2.20– 3.06 | <0.001 |
| **Study year** |  |  |  |
| 2002/2003 | 1.0 |  |  |
| 2004/2005 | 1.09 | 0.93 – 1.27 | 0.295 |
| 2006/2007 | 1.27 | 1.09 – 1.48 | 0.002 |
| 2008/2009 | 1.18 | 1.01 – 1.38 | 0.037 |
| **Study site** |  |  |  |
| Community Hospitals | 1.0 |  |  |
| Tertiary Hospitals | 1.08 | 0.96 – 1.21 | 0.221 |
| **ICU LOS (log)** | 1.27 | 1.19 – 1.36 | <0.001 |
| **Admission diagnosis** |  |  |  |
| Respiratory | 1.0 |  |  |
| Gastrointestinal | 1.87 | 1.60 – 2.21 | <0.001 |
| Cardiovascular | 1.32 | 1.11 – 1.57 | 0.002 |
| Sepsis | 1.17 | 0.97 – 1.41 | 0.091 |
| Trauma | 0.85 | 0.63 – 1.15 | 0.296 |
| Metabolic | 0.54 | 0.38 – 0.76 | <0.001 |
| Neurologic | 2.20 | 1.80 – 2.68 | <0.001 |
| Renal | 1.11 | 0.82 – 1.47 | 0.498 |
| Other | 1.77 | 1.26 – 2.47 | 0.001 |

Abbreviations: OR = odds ratio; ED = emergency department; LOS: length of stay

AuROC: 0.787 (0.777 – 0.797)

GoF test: 0.991
